# msBERT-Promoter: a multi-scale ensemble predictor based on BERT pre-trained model for the two-stage prediction of DNA promoters and their strengths

**DOI:** 10.1186/s12915-024-01923-z

**Published:** 2024-05-30

**Authors:** Yazi Li, Xiaoman Wei, Qinglin Yang, An Xiong, Xingfeng Li, Quan Zou, Feifei Cui, Zilong Zhang

**Affiliations:** 1 School of Mathematics and Statistics, Hainan University, Haikou, 570228 China; 2 School of Computer Science and Technology, Hainan University, Haikou, 570228 China; 3 Institute of Fundamental and Frontier Sciences, University of Electronic Science and Technology of China, Chengdu, 610054 China; 4 Yangtze Delta Region Institute (Quzhou), University of Electronic Science and Technology of China, Quzhou, 324000 China

**Keywords:** DNA promoters, Pre-trained model, BERT, Soft voting, Two-stage predictor

## Abstract

**Background:**

A promoter is a specific sequence in DNA that has transcriptional regulatory functions, playing a role in initiating gene expression. Identifying promoters and their strengths can provide valuable information related to human diseases. In recent years, computational methods have gained prominence as an effective means for identifying promoter, offering a more efficient alternative to labor-intensive biological approaches.

**Results:**

In this study, a two-stage integrated predictor called “msBERT-Promoter” is proposed for identifying promoters and predicting their strengths. The model incorporates multi-scale sequence information through a tokenization strategy and fine-tunes the DNABERT model. Soft voting is then used to fuse the multi-scale information, effectively addressing the issue of insufficient DNA sequence information extraction in traditional models. To the best of our knowledge, this is the first time an integrated approach has been used in the DNABERT model for promoter identification and strength prediction. Our model achieves accuracy rates of 96.2% for promoter identification and 79.8% for promoter strength prediction, significantly outperforming existing methods. Furthermore, through attention mechanism analysis, we demonstrate that our model can effectively combine local and global sequence information, enhancing its interpretability.

**Conclusions:**

msBERT-Promoter provides an effective tool that successfully captures sequence-related attributes of DNA promoters and can accurately identify promoters and predict their strengths. This work paves a new path for the application of artificial intelligence in traditional biology.

**Supplementary Information:**

The online version contains supplementary material available at 10.1186/s12915-024-01923-z.

## Introduction

A promoter is a specific DNA sequence that initiates transcription and controls the timing and location of gene expression in an organism [1]. One common promoter sequence in eukaryotic genes is the TATA box, which attracts transcription factors, leading to the formation of RNA polymerase transcription complexes and the initiation of transcription [2]. Promoters in eukaryotic cells can vary in length (100–1000 base pairs) and are categorized into three main types: proximal promoters, distal promoters, and core promoters. These promoter types play distinct roles in DNA transcription and the activity of RNA polymerase. Extensive research shows that disruptions in promoter function can lead to a range of diseases, such as gastric cancer [3] and B cell lymphoma [4], by affecting gene expression. Identifying promoters is crucial for understanding gene expression regulation as they often cooperate with regulatory elements via chromatin loops and play important roles in developmental diseases, tumorigenesis, and spatiotemporal gene expression [5–9]. However, accurately predicting promoters remains a challenging task.

With the rapid development of next-generation sequencing tools, biologists can use them for related research, with the main methods being RNA polyadenylation (5′-phosphate phosphatase) sequencing (PPP-seq) [10], Cappable-seq [11], and chromatin immunoprecipitation sequencing (ChIP-seq) [12]. However, most wet lab experiments are expensive and time-consuming, and with the exponential growth of biological sequences in the post-genomic era, it is necessary to propose computational methods to address the issue [13–17].

In order to address the issue, several computational models have been developed in the past decade for early prediction of promoters. For example, Lin et al. [18] utilized support vector machine (SVM) and pseudo k-tuple nucleotide composition (PseKNC) to identify δ_24_ promoters in prokaryotes. iPromoter-2L [19] is a two-layer promoter predictor that employs multi-window pseudo k-tuple nucleotide composition to distinguish promoters from non-promoters and classify six types of promoters. iProEP [20] predictor constructs a feature matrix using the PseKNC and position-correlation scoring function (PCSF) methods. It then utilizes the increment feature selection strategy and minimum redundancy maximum relevance (mRMR) algorithm to search for the optimal feature subset, followed by SVM classification to discriminate promoters from non-promoters. MULTiPly [21] is a multi-layer approach that combines local information, such as k-tuple nucleotide composition, with global information encoded by dual-profile Bayesian and K-nearest neighbor features. It employs the *F*-score method for feature selection and utilizes SVM for prediction. Although the aforementioned methods are indeed capable of identifying promoters, they heavily rely on traditional feature encoding techniques and machine learning models. These approaches often necessitate intricate feature engineering steps and lack the incorporation of contextual semantic relationships.

In recent years, Bidirectional Encoder Representations from Transformers (BERT) [22] has demonstrated outstanding performance in various natural language processing (NLP) tasks [23]. Due to the similarity between biological sequence data and textual data, it also shows promising results in biological scenarios [24, 25]. For example, the PreRBP-TL [26] model incorporates self-attention mechanisms in its architecture to enhance the recognition of RNA-binding proteins (RBPs). This approach enables more effective feature learning and identification, improving the accuracy of predicting RBPs across different species. miProBERT [27] utilizes fine-tuned BERT for accurate identification of microRNA promoters, outperforming other prediction methods for gene promoters. RBP-TSTL [28] utilizes a two-stage transfer learning framework for the genome-scale prediction of RNA-binding proteins, effectively leveraging self-attention mechanisms to improve prediction accuracy. Rm-LR [29] integrates local and global information through bilinear attention networks to accurately predict various types of RNA modifications, achieving state-of-the-art results on eight RNA modification datasets. These research achievements demonstrate the powerful ability of BERT to capture complex patterns in sequence data, making it an effective benchmark model.

Promoters can be categorized as strong promoters or weak promoters based on their levels of transcriptional activation and expression. Accurately predicting promoter strength is essential for comprehending gene transcription regulation mechanisms and constructing expression regulatory networks. Hence, besides identifying promoters, predicting their strength is also significant. In recent years, several classification methods have been proposed for predicting promoter strength. For instance, Le et al. [30] interprets DNA sequences as combinations of continuous FastText N-grams and classifies them using deep neural networks. BERT-Promoter [31] combines the BERT model with Shapley Additive exPlanations (SHAP) [32] analysis for feature extraction and uses random forests for classification, achieving good performance in both promoter identification and promoter strength prediction. iPromoter-CLA [33] employs deep capsule networks and bidirectional long short-term memory networks to identify promoters and their strengths in DNA sequences. Although these methods can identify promoters and predict their strengths, there is still room for improvement in terms of prediction accuracy.

Previous studies mainly used pre-trained models to extract features and simply input them into the model for prediction, without exploring the strategy of ensemble learning for pre-trained models. However, ensemble learning often exhibits better performance than the base models. In our research, we propose a novel predictor called “msBERT-Promoter,” which is a two-stage predictor with the first stage used for promoter classification and the second stage for predicting promoter strength. The basic framework we adopt is the BERT pre-trained model. By employing different tokenization strategies, the original sequence is divided into tokens of varying lengths to integrate local and global information. These tokenized sequences are then encoded to form different feature matrices, which are subsequently fed into BERT layers to extract potential information between sequences. To obtain more reliable results, we use a soft voting ensemble method to combine the predictions of different base models and study the interpretability of the model through visual attention heatmaps. Our experiments on prokaryotic promoter datasets assessed the algorithm’s performance. The first layer of our model achieved an ACC of 96.2%, a MCC of 0.923, and an AUC of 0.994. In the second layer, the model predicted promoter strength with an ACC of 79.8%, a MCC of 0.595, and an AUC of 0.874. The prediction accuracy of both layers surpasses that of state-of-the-art predictors for promoter identification and strength prediction within the same dataset.

## Materials and methods

### Benchmark dataset

Selecting an appropriate baseline dataset for training and testing the model is crucial in developing effective predictors for application in biological sequences. In this study, we evaluated the performance of our model using the benchmark dataset of the iPSW (2L)-PseKNC [34] method. RegulonDB (version 9.4) [35] is a database containing information on the transcriptional regulatory network of Escherichia coli and is one of the most commonly used resources in the field of bacterial gene regulation research. To train and test our model, we need to collect the required promoter sequences from RegulonDB. A promoter is a DNA regulatory region approximately 100–1000 base pair long, from which we selected 81 bp core promoter sequences as the input samples.

**Fig. 1:**
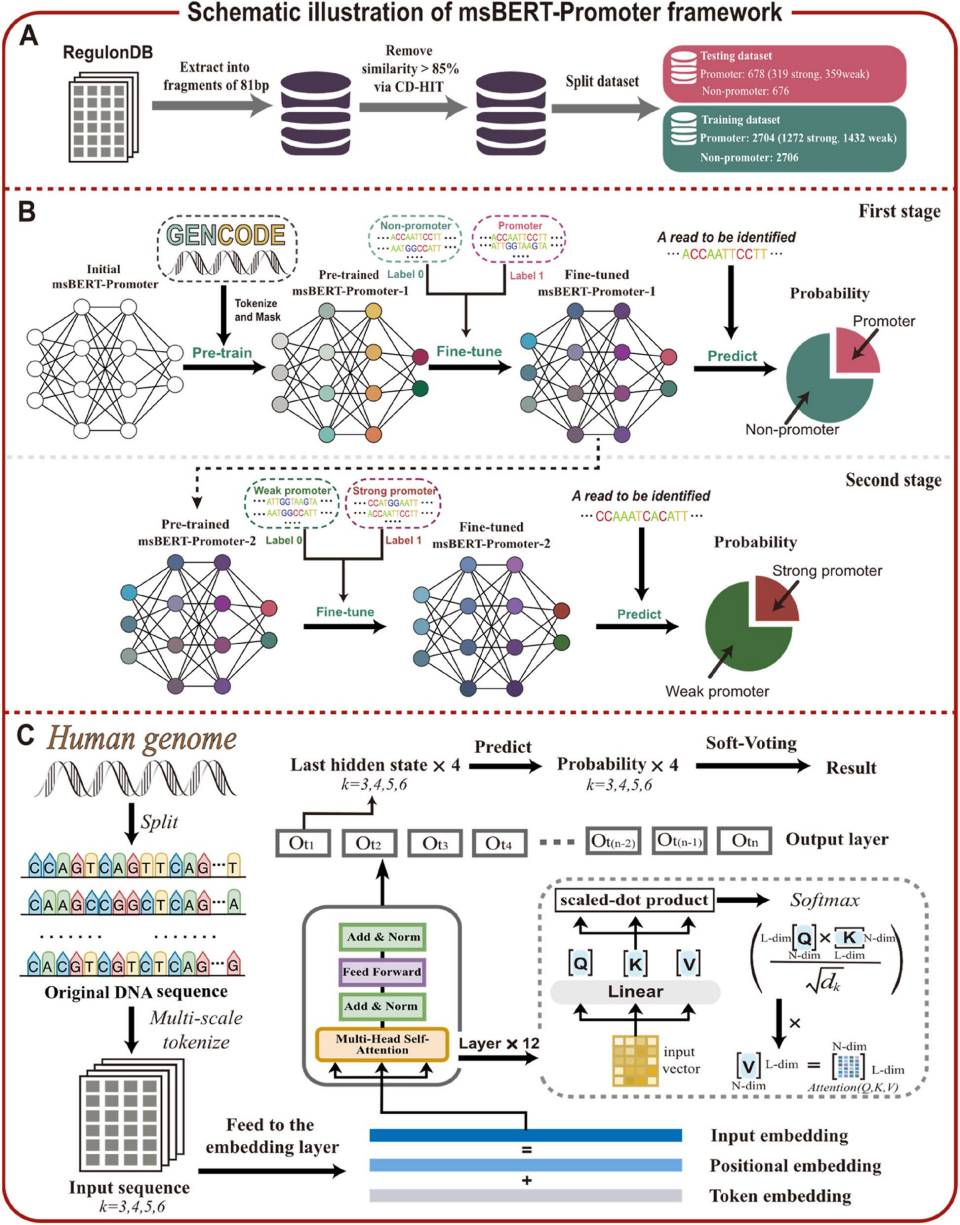
Schematic illustration of msBERT-Promoter framework. **A** Constructing benchmark datasets. **B** Two-stage ensemble classifier explanation. In the first stage, BERT is first pre-trained on a large corpus of text data, then fine-tuned on a specific task using labeled data, and finally used for making predictions. The model obtained after fine-tuning in the first stage serves as the pre-trained model for the second stage, where further fine-tuning is performed. **C** Detailed process from fine-tuning to prediction. Firstly, relevant DNA segments are extracted from the human genome as the original DNA sequence. These sequences are then tokenized into tokens of lengths 3, 4, 5, and 6, which are fed into an embedding layer. The multi-head self-attention mechanism is applied to extract text information from each token length separately and make predictions. Finally, the soft voting ensemble strategy is employed to obtain the final prediction by combining the predictions from different token lengths

To eliminate redundancy in the samples, we must cluster highly similar sequences to retain representative sequences. For this purpose, we used CD-HIT [36] to remove sample fragments with similarity greater than 85%, resulting in 3382 core promoter samples being retained as the positive sample dataset. In order to perform a binary classification task, we extract 3382 81-bp sequence fragments from the non-core promoter dataset to form the negative sample dataset. These sample datasets together constitute the benchmark dataset. The benchmark dataset was then divided into testing and training datasets with a ratio of 1:4. Finally, as shown in Fig. 1A, the training dataset contains 2704 promoter samples (1272 strong promoters and 1432 weak promoters) and 2706 non-promoter samples, while the testing dataset contains 678 promoter samples (319 strong promoters and 359 weak promoters) and 676 non-promoter samples.

### The BERT model

BERT is a bidirectional natural language processing (NLP) model based on the Transformers structure. Unlike traditional Transformers that consist of multiple encoders and decoders, BERT only retains the encoder part. BERT inherits the multi-head attention mechanism and feed-forward neural network from Transformers, while also adding the function of bidirectional learning.[37]. This feature enables the BERT model to mine deeper contextual semantic information and achieve state-of-the-art performance in multiple NLP tasks.

To build a universal pre-training model, BERT adopts two training tasks, namely next sentence prediction (NSP) and masked language model (MLM). In the MLM task, the sentence needs to be converted into token representation. Then, a special token [CLS] is added at the beginning of each sentence to capture the entire sequence information. Another special token [SEP] is added at the end of the sentence to separate different sentences and understand the boundaries and correlations of multiple sentence inputs. Then, 15% of the tokens in a sentence are randomly masked with the [MASK] token, and the model is trained to predict these masked words from the remaining sentence based on the context. However, since many downstream tasks depend on analyzing the relationship between two sentences to model, a binary classification task for predicting the next sentence is proposed to enable the model to have this ability. To meet these two requirements, the embedding layer of BERT includes three layers of information: token embedding, position embedding, and segment embedding. Token embedding converts tokenized sentences into fixed-dimensional vectors, position embedding identifies the position of each token in the sentence, and segment embedding distinguishes the different positions of the two sentences, mainly used in the NSP task. The results of these three parts are added and normalized to obtain the final input embedding. Then, after capturing the contextual information of the current position through multi-head attention mechanism and reducing the risk of overfitting through residual connections and layer normalization, the input is transformed linearly in the Feed Forward to extract deeper features.

### Pre-training of DNABERT

DNABERT [38] follows the training process of BERT but removes the NSP training task to develop a pre-training model specifically for genomic sequences. To accommodate biological contexts, DNABERT treats *k* consecutive nucleotides as a group, known as a k-mer, and chooses *k* values of 6, 5, 4, and 3. The initial input requested by DNABERT consists of a set of sequences represented as k-mer tokens. As a result, each token is transformed into a numeric vector, and each sequence is represented as a matrix. These matrices are then fed into a multi-head self-attention mechanism to capture contextual information. The relevant formulas are as follows:

1 $$\text{Multi-Head}\left( Q,K,V\right)={\text{Concat}}\left(\text{ hea}{\text{d}}_{1},\dots ,{\text{hea}}{\text{d}}_{\text{h}}\right){ W}^{o}$$

where

2 $$\text{head}_{i}=\text{Attention}\left(Q{W}_{i}^{Q},K{W}_{i}^{K},V{W}_{i}^{V}\right)$$

3 $$\text{Attention}(Q,K,V)=\text{softmax}\left(\frac{{QK}^{T}}{\sqrt{{d}_{k}}}\right)V$$

In the above formulas, “*head*_*i*_” refers to the attention layer, and “*Q*” represents the query vector, which measures the degree of association between the current position and other positions. “*K*” represents the key vector, which is used to measure attention allocation. “*d*_*k*_” represents the dimensionality of the vectors. “*V*” represents the value vector, which is weighted and summed based on the association between “*K*” and “*Q*.” The matrix multiplication of the two matrices, “*Q*” and “*K*^*T*^,” yields the attention scores between the word vectors and other positions. These attention scores are then transformed into a probability distribution using the softmax function, where the sum of probabilities is 1. The probability indicates the magnitude of correlation between the word vector and another word, with values closer to 1 indicating a stronger correlation. Finally, the result is multiplied by “*V*” to obtain the new encoded vector for that position.

Since DNABERT in this study consists of 12 encoding layers, the attention mechanism is executed 12 times, resulting in 12 heads as output. These 12 results are concatenated together and compressed using a linear transformation matrix “*W*^*O*^” to form a fully connected layer, which serves as the input for the next prediction step. The fully connected layer not only reduces the dimensionality of the feature vectors but also greatly enhances robustness. The parameters used in this process are not shared, allowing each head to independently learn different features. Therefore, the multi-head attention mechanism can learn more comprehensive contextual relationships while ensuring operational efficiency. Finally, four pre-trained models were obtained: DNABERT-6mer, DNABERT-5mer, DNABERT-4mer, and DNABERT-3mer.

### Fine-tune of DNABERT

In this study, we fine-tuned the aforementioned four pre-trained models to adapt them to specific task scenarios. Firstly, the dataset was split into four different formats: 6-mer, 5-mer, 4-mer, and 3-mer, by dividing each 81 bp-long sequence. The promoter dataset and non-promoter dataset were then separately inputted into the pre-trained models for fine-tuning, resulting in four base models in the first layer: DNABERT-6mer-1, DNABERT-5mer-1, DNABERT-4mer-1, and DNABERT-3mer-1. Subsequently, the strong promoter and weak promoter data were further inputted into the four base models obtained in the first layer for additional fine-tuning, resulting in four base models in the second layer: DNABERT-6mer-2, DNABERT-5mer-2, DNABERT-4mer-2, and DNABERT-3mer-2.

### Soft voting ensemble method

In the previous context, the pre-trained DNABERT models were fine-tuned to obtain eight fine-tuned pre-trained models. To make more accurate predictions, we employed ensemble learning to combine these pre-trained models. For the classification task, we chose the soft-voting classifier, which combines and votes on the prediction results generated under the conditions of *k* = 3, 4, 5, and 6. Soft-voting ensemble learning requires that the prediction results of each model in the ensemble can be transformed into probability values. For the probability values of each class, a weighted average is calculated, and its mathematical formula is as follows:

4 $$H\left(x\right)={\text{argmax}}{ }_{\text{j}}\left(\sum\limits_{i=1}^{T}{w}_{i}^{j}\times {h}_{i}^{j}\left(x\right)\right),{h}_{i}^{j}\left(x\right)\in \left[\text{0,1}\right]$$

In the formula, *T* represents the number of base classifiers ${h}_{i}$, and* j* represents a certain class of input data. ${\omega }_{i}^{j}$ represents the weight of the *j*th class input for the base classifier ${h}_{i}$, where ${\omega }_{i}^{j}$ takes values in the range [0, 1]. ${h}_{i}^{j}$*(x)* represents the probability estimate of the base classifier ${h}_{i}$ for the *j*th class input, where ${h}_{i}^{j}$*(x)* takes values in the range [0, 1]. The weight ${\omega }_{i}^{j}$ is multiplied by ${h}_{i}^{j}$*(x)* to obtain the proportion probability estimate of the *i*th classifier for the *j*th class input among the *T* base classifiers. After summing up these *T* products, we obtain the weighted average probability for a single input. Since the input data, as the independent variable, can be divided into *j* classes, we can obtain *j* weighted average values. Then, the argmax function is applied to determine the class corresponding to the maximum value, which is output as the final result.

### Performance evaluation metrics

To evaluate the performance of the model, we used five commonly used evaluation metrics, including accuracy (ACC), sensitivity (Sn), specificity (Sp), Matthews correlation coefficient (MCC), and area under the receiver operating characteristic curve (AUC) [39–43]. The formulas for these metrics are as follows:

5 $${\text{Sn}}=\frac{\text{TP}}{{\text{TP}}+{\text{FN}}}$$

6 $${\text{Sp}}=\frac{\text{TN}}{{\text{TN}}+{\text{FN}}}$$

7 $${\text{ACC}}=\frac{{\text{TP}}+{\text{TN}}}{{\text{TP}}+{\text{TN}}+{\text{FP}}+{\text{FN}}}$$

8 $${\text{MCC}}=\frac{{\text{TP}}\times {\text{TN}}-{\text{FP}}\times {\text{FN}}}{\sqrt{\left({\text{TP}}+{\text{FP}}\right)\times \left({\text{TP}}+{\text{FN}}\right)\times \left({\text{TN}}+{\text{FP}}\right)\times \left({\text{TN}}+{\text{FN}}\right)}}$$

where TP, TN, FN, and FP represent the numbers of true positives, true negatives, false negatives, and false positives, respectively. Sn represents the proportion of positive samples correctly identified. Sp represents the proportion of negative samples correctly identified. ACC represents the proportion of all samples that are correctly classified. MCC measures the correlation between the true values and the predicted values, with a range of [− 1, 1]. Additionally, to comprehensively compare the performance of different models, it is necessary to calculate the evaluation metric based on the area under the receiver operating characteristic (ROC) curve, which displays the ratio of true positives to false positives. The AUC value ranges from 0 to 1, with a higher AUC indicating better predictive performance of the underlying model. In general, higher values of these five metrics indicate better model performance.

### Experimental setup

Experiments were conducted using an NVIDIA GeForce GTX 4090 with 24 GB memory. Adam optimizer with a 0.01 weight decay was used to update model parameters. A linear schedule with a 0.1 warmup percent was used to decrease the learning rate linearly after linearly increasing during a warmup period. The maximum sequence length was set to 81. The training phase was bounded by a maximum of 100 epochs, with an early stopping mechanism in place to halt the training if no discernible improvement in AUC was observed over a period of ten consecutive epochs. Additional files 1: Tables S1 and S2 provide a comprehensive overview of the detailed hyperparameter configurations.

## Results and discussion

### Comparison with the baseline predictors on promoter classification and promoter strength classification

**Fig. 2:**
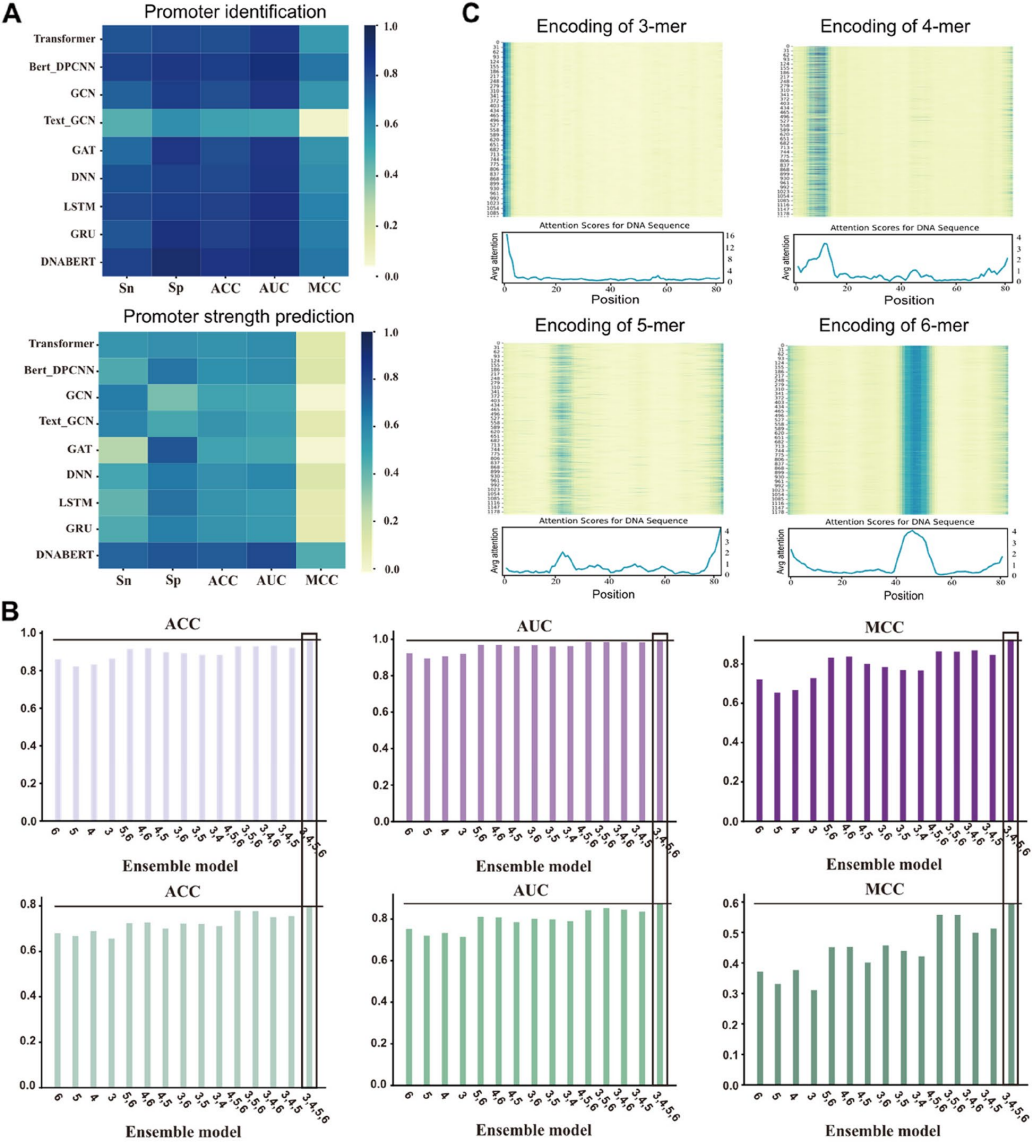
**A** Comparison of prediction performance of eight baseline predictors in promoter identification and promoter strength prediction. **B** Performance comparison of ensembles of different base models. The top three figures show the prediction results for promoter identification, while the bottom three figures show the prediction results for promoter strength prediction. **C** Attention map of four encoding schemes, including 3-mer, 4-mer, 5-mer, and 6-mer, respectively

To demonstrate the superiority of DNABERT, we compared it with several typical deep learning models, including Transformer [44], Bert_DPCNN [45], GCN [46], Text_GCN [47], GAT [48], DNN [49], LSTM [50], and GRU [51]. The architectures of these deep learning models were implemented by the DeepBIO server [52] to ensure a fair comparison. As shown in Fig. 2A, in terms of promoter classification, DNABERT outperformed all typical pretrained models and deep learning methods in terms of Sn, Sp, ACC, AUC, and MCC metrics. For example, compared to Transformer, DNABERT achieved a 9.6% improvement in ACC and an 11.7% improvement in MCC. Compared to Bert_DPCNN, DNABERT showed a 3.4% improvement in ACC. Furthermore, compared to GAT, DNABERT demonstrated a 9% improvement in ACC. In terms of promoter strength classification, DNABERT outperformed Transformer by 16.7% in ACC and 33.3% in MCC. Compared to Bert_DPCNN, DNABERT achieved a 15.9% improvement in ACC. Additionally, compared to GAT, DNABERT showed a 22.4% improvement in ACC (refer to the Additional files 2: Table S3 and S4 for detailed data).

It is worth noting that compared to other deep learning methods, graph-based deep learning methods generally exhibit poorer performance. This is because DNA sequences often do not contain inherent graph-related information and therefore cannot be properly represented as graphs. This indirectly introduces some noise, which interferes with prediction. On the other hand, common approaches for processing natural language have achieved better performance, such as Transformer, LSTM, and GRU. This is because biological sequences have a great similarity to natural language in essence. Therefore, processing biological sequence data like natural language often yields better results. Among the methods for processing natural language, attention-based methods often achieve better performance due to their powerful ability to understand context. In addition, DNABERT, having been pre-trained on a large amount of biological data, tends to exhibit superior performance in biological scenarios compared to other attention-based methods. In conclusion, these results indicate that DNABERT effectively harnesses the potential of pretrained models. It exhibits superior predictive capability for promoter sequences compared to typical pretrained models and deep learning methods.

### Comparison with previously published predictors on benchmark dataset

In order to demonstrate the effectiveness of our model, we compared it with other state-of-the-art predictors on the same benchmark dataset, including Le et al. [30], iPSW (PseDNC-DL) [53], BERT-Promoter [31], iPSW(2L)-PseKNC [34], and iPromoter-CLA [33]. Among the predictors mentioned above, iPSW (PseDNC-DL) and iPSW(2L)-PseKNC focused on solving the problem by utilizing optimal features based on nucleotide compositions. Meanwhile, Le et al. attempted to address the issue by using a combination of fastText model and convolutional neural network (CNN). iPromoter-CLA uses a combination of capsule neural network and recurrent neural network (RNN) to identify promoters and their strengths. In comparison, our model msBERT-Promoter eliminates the need for complex feature encoding projects required by the aforementioned methods. Moreover, most of the above methods use traditional machine learning or deep learning method, without using self-attention mechanism to understand semantic relationship within the sequence. To ensure fairness, we conducted a comparison experiment using the same dataset and evaluation metrics.

Our model outperformed previous models in terms of ACC, Sn, ROC, and MCC for both first-stage promoter recognition and second-stage promoter strength in the independent test dataset. As seen in Table 1, all indicators have achieved good performance, among which achieved a specificity of 0.951, sensitivity of 0.973, accuracy of 0.962, AUC of 0.994, and MCC of 0.923 in the first layer, whereas the second layer achieved the average specificity of 0.786, sensitivity of 0.814, accuracy of 0.798, AUC of 0.874, and MCC of 0.595. These results demonstrated the effectiveness of our proposed model in promoter identification and promoter’s strength classification.

**Table 1:** Comparison to previously published predictors

Predictors	Sn	Sp	Acc	AUC	MCC
1st layer					
iPSW(2L)-PseKNC	0.814	0.849	0.831	0.905	0.663
Le et al	0.828	0.881	0.854	/	0.709
iPSW (PseDNC-DL)	0.833	0.868	0.851	0.925	0.702
BERT-Promoter	0.843	0.866	0.855	/	/
iPromoter-CLA	0.869	0.851	0.860	0.929	0.721
Ours	0.973	0.951	0.962	0.994	0.923
2nd layer					
iPSW(2L)-PseKNC	0.622	0.792	0.712	0.776	0.421
Le et al	0.694	0.764	0.731	/	0.460
iPSW (PseDNC-DL)	0.658	0.782	0.724	0.790	0.444
BERT-Promoter	0.709	0.816	0.769	/	/
iPromoter-CLA	0.776	0.688	0.735	0.796	0.470
Ours	0.814	0.786	0.798	0.874	0.595

It is noteworthy that the features extracted by deep learning generally outperform traditional handcrafted features, as we have observed in Table 1. In common deep learning methods, utilizing attention mechanisms often leads to better performance. This is one of the reasons why BERT-Promoter and iPromoter-CLA methods outperform previously proposed methods. With the rapid development of large language models, pre-trained models in biological contexts often demonstrate superior performance in biological sequence classification problems. Through unsupervised learning on biological data, these models can enhance their understanding of biological data, thereby exhibiting better performance in downstream tasks related to biology.

### Ablation experiment identified the effectivity of msBERT-Promoter

Firstly, we conducted ablation experiments to demonstrate the effectiveness of the soft voting ensemble method. We systematically explored all possible combinations and observed that as the number of base models decreased, the performance of these models weakened to varying degrees. As we can see in Fig. 2B, in terms of promoter identification, there was a decrease in model accuracy by 3–14%, AUC by 1–10%, and MCC by 6–26%. For promoter strength prediction, the model’s accuracy decreased by 4–14%, AUC by 2–16%, and MCC by 4–28%. These results indicate that msBERT-Promoter effectively integrates the predictive performance of diverse base models through the soft voting ensemble strategy, resulting in a more robust and high-performing integrated model.

Consequently, in order to validate the efficacy of sequential connectivity in two-stage fine-tuning, we conducted an additional set of experiments. Specifically, we performed fine-tuning on both the promoter identification dataset and the promoter strength prediction dataset separately, denoting them as msBERT-Promoter-X. The experimental outcomes are detailed in Table 2. Notably, in the realm of promoter strength prediction, the predictive performance of msBERT-Promoter surpassed that of msBERT-Promoter-X. This observation underscores the capacity of sequential connectivity to leverage insights acquired during the initial fine-tuning stage to enhance comprehension of the subsequent task, resulting in a 6.63% enhancement in prediction accuracy, a 5.05% increase in AUC, and a notable 12.88% rise in MCC.

**Table 2:** Ablation study of the two-stage prediction scheme

Predictors	Sn	Sp	Acc	AUC	MCC
1st layer					
msBERT-Promoter-X	0.9728	0.9509	0.9616	0.9943	0.9234
msBERT-Promoter-Y	0.9538	0.9384	0.9485	0.9832	0.9029
ours	0.9728	0.9509	0.9616	0.9943	0.9234
2nd layer					
msBERT-Promoter-X	0.7796	0.7044	0.7316	0.8237	0.4658
msBERT-Promoter-Y	0.7796	0.7044	0.7316	0.8237	0.4658
ours	0.8138	0.7861	0.7979	0.8742	0.5946

Moreover, to corroborate the validity of the sequence in sequential connectivity, we executed a secondary set of experiments. Initially, we fine-tuned the promoter strength prediction dataset, followed by inputting the promoter identification dataset into the previously fine-tuned model, denoted as msBERT-Promoter-Y. As delineated in Table 2, across both the promoter identification and promoter strength prediction stages, the predictive performance of msBERT-Promoter consistently outperformed that of msBERT-Promoter-Y. This discrepancy can be elucidated from dual perspectives. Primarily, regarding promoter strength prediction, the absence of prior enrichment with promoter identification data hindered the profound understanding of promoter sequence data by msBERT-Promoter-Y, thereby impeding its capability to delve deeply into the task of predicting promoter strength. Subsequently, in the context of promoter identification, the constrained comprehension of promoter data during the initial stage of learning promoter strength prediction tasks in msBERT-Promoter-Y might have engendered negative feedback within its learned experience, potentially hampering its assimilation of promoter identification data and consequently resulting in inferior predictive performance compared to msBERT-Promoter.

In summary, the results derived from this series of experiments underscore the robust rationale and superior performance of our model.

### Attention mechanism analysis

To improve the interpretability of the model and pinpoint crucial sequence sites for identifying promoters and predicting their strength, we performed an attention mechanism analysis, visualizing the attention weights of various tokenizer schemes. From Fig. 2C, it can be observed that the high attention weight regions for the four models on the same sample are at positions 1–4, 7–13, 80–81, and 43–51. This indicates that they capture completely different sequence information through different tokenization schemes. Shorter sequence fragments provide the models with a large amount of local information but lack relevant global information. On the contrary, longer sequence fragments grasp broader global information. Different input lengths result in changes in the positions of key features in the encoded sequences, which in turn cause variations in attention distribution.

Therefore, it is crucial to effectively integrate the information extracted from different approaches. To achieve this, we designed several sets of experimental schemes. First is by directly adding all extracted features and then feeding them into a fully connected layer for prediction (called as Ensemble_A). To ensure fairness, all parameters of the fully connected layer are kept consistent with DNABERT’s default parameters. Since directly adding all features may lead to feature redundancy, in the second set of experiments, we incorporated a feature selection algorithm. We used the LightGBM algorithm to rank the added features based on importance and selected 782-dimensional features with importance greater than 0. Subsequently, the selected features were inputted into the fully connected layer for prediction (called as Ensemble_B). As shown in Table 3, experimental results indicate that directly adding features extracted by all base predictors can indeed enhance prediction performance. Moreover, after introducing the feature selection step, the redundancy among features was somewhat alleviated. However, the final performance still did not surpass that achieved using a soft voting ensemble strategy. This is because operations at the feature level often lead to feature redundancy or insufficient feature information. In contrast, the soft voting ensemble strategy integrates at the final prediction level, which avoids compromising the final performance due to feature redundancy, thus demonstrating better performance.

**Table 3:** Ablation study of the soft voting ensemble scheme on promoter identification and promoter strength prediction

Predictors	Promoter identification					Promoter strength prediction				
	Sn	Sp	Acc	AUC	MCC	Sn	Sp	Acc	AUC	MCC
DNABERT_3mer	0.8718	0.8567	0.8641	0.92	0.7284	0.6319	0.6790	0.6563	0.7139	0.3112
DNABERT_4mer	0.8641	0.8057	0.8323	0.907	0.6672	0.7195	0.6713	0.6888	0.7333	0.3765
DNABERT_5mer	0.8854	0.7776	0.8227	0.8943	0.6542	0.6679	0.6683	0.6681	0.7210	0.3316
DNABERT_6mer	0.8896	0.834	0.8597	0.9230	0.7215	0.6328	0.7415	0.6799	0.7530	0.3716
Ensemble_A	0.9169	0.8953	0.9023	0.9523	0.8437	0.7209	0.7623	0.7487	0.8102	0.4923
Ensemble_B	0.9241	0.9417	0.9318	0.9718	0.8901	0.7984	0.7591	0.7742	0.8542	0.5428
Soft voting (ours)	0.9728	0.9509	0.9616	0.9943	0.9234	0.8138	0.7861	0.7979	0.8742	0.5946

In summary, through attention analysis, we provide insights into the interpretability of the models and emphasize the importance of utilizing a soft voting ensemble strategy to integrate different base learners for improving promoter identification and promoter strength prediction.

### The interpretability analysis of soft voting ensemble strategy

**Fig. 3:**
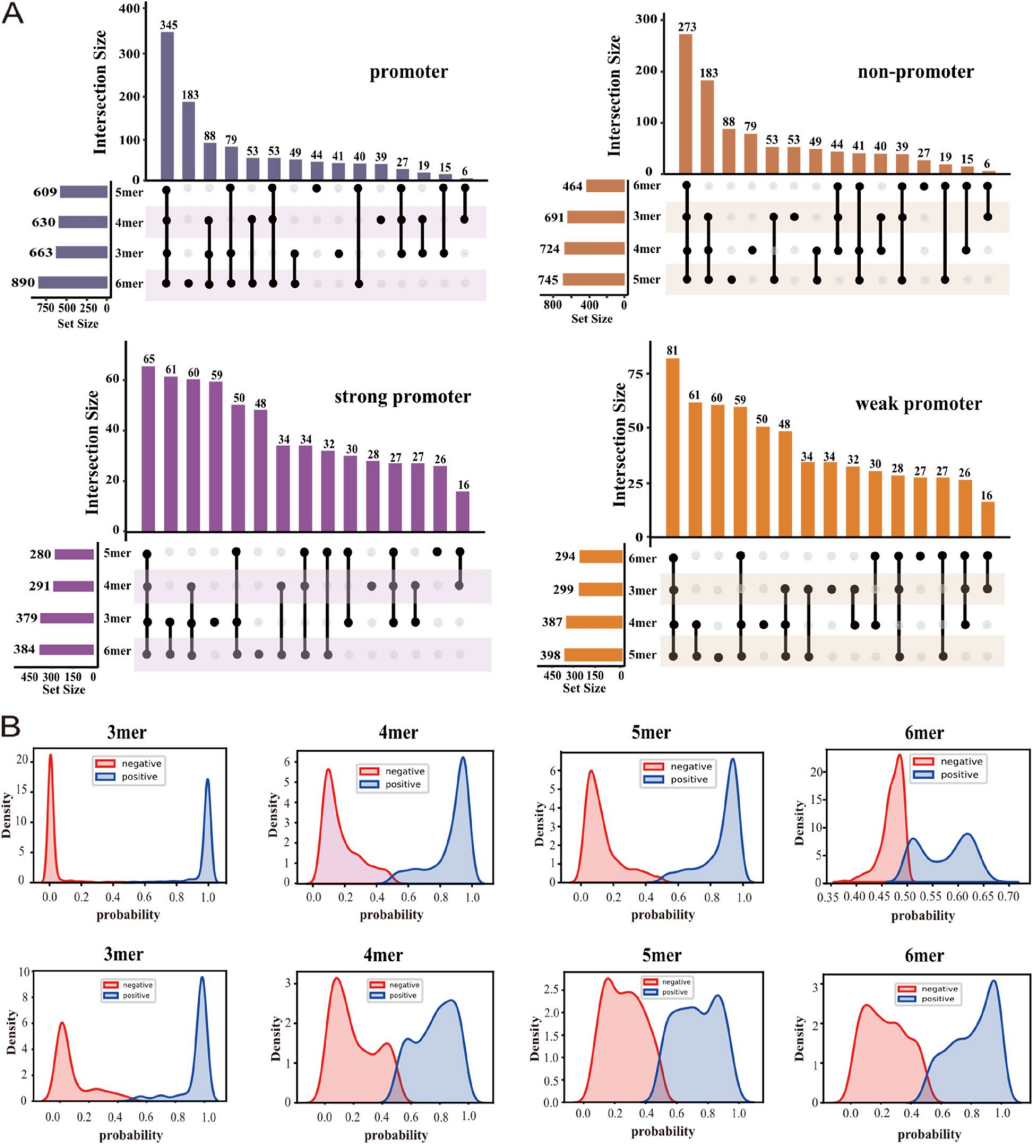
The interpretability analysis of soft voting ensemble strategy. **A** The UpSet plot visualizes the intersection of the predictive results from four base models for promoter, non-promoter, strong promoter, and weak promoter. Among them, the horizontal bar chart represents the number of elements contained in different sets, while the vertical bar chart represents the number of elements contained in the intersections of different sets. The black dots connected by black vertical lines indicate which sets are intersecting. **B** The kernel density estimation plot visualizes the probability distribution of predictions from four base models for promoter, non-promoter, strong promoter, and weak promoter, with the top four representing the first stage and the bottom four representing the second stage

In order to gain a more intuitive understanding of how soft voting contributes to improving prediction accuracy, a series of visualizations were implemented. Firstly, an UpSet plot was used to describe in detail the prediction distribution of each base predictor in terms of promoter, non-promoter, strong promoter, and weak promoter. As shown in Fig. 3A, in the first stage (promoter identification stage), 1354 samples were tested, where 345 samples were predicted as promoters and 273 samples were predicted as non-promoters by all four base predictors simultaneously. These samples cannot have their predicted labels changed by the soft voting ensemble strategy; hence, they do not affect the final prediction performance. Additionally, there were 736 samples predicted differently by the base predictors (as promoters or non-promoters), which can potentially improve the final prediction performance through soft voting. Similarly, in the second stage (promoter strength prediction stage), out of 678 tested samples, 65 and 81 samples were predicted as strong promoters or weak promoters by all four base predictors simultaneously. These samples cannot have their final predictions changed by soft voting, but 532 samples predicted differently by the base predictors (as strong promoters or weak promoters) can potentially improve the overall prediction accuracy.

After analyzing that the soft voting strategy has the potential to enhance overall prediction performance, we further explored how the soft voting strategy improves the model’s overall performance by visualizing the kernel density estimates of prediction probabilities for all samples by eight base learners.

As shown in Fig. 3B, in the first stage, the 3-mer, 4-mer, and 5-mer base predictors effectively differentiate between positive and negative samples. The prediction probabilities for positive samples are mainly concentrated in the range of 0.05–0.15, while for negative samples, they are mainly concentrated in the range of 0.95–0.98. This demonstrates the model’s excellent ability to discriminate between different sample classes. However, the 6-mer base predictor does not clearly distinguish between positive and negative samples. The prediction probabilities for positive samples are mainly around 0.47, while for negative samples, they are concentrated in the range of 0.5–0.52 and 0.61–0.63. Despite the 6-mer base predictor showing similar performance to other base predictors, it can be further improved through the soft voting ensemble strategy. By combining the prediction probabilities of the four base predictors and leveraging the strengths of the 3-mer, 4-mer, and 5-mer predictors in terms of prediction probability distribution, the weaknesses of the 6-mer predictor can be compensated for, leading to an 11–14% improvement in promoter identification accuracy. Conversely, although the 3-mer, 4-mer, and 5-mer predictors can effectively differentiate between different sample classes, there are still some samples that are difficult to classify correctly. The 6-mer base predictor can assist in improving the overall prediction performance on these minority samples, demonstrating the collaborative role of each base predictor. In the second stage, all four base predictors can differentiate to a certain degree between positive and negative samples, but there are still some samples that are not properly classified, and the number of misclassified samples is relatively higher compared to the first stage due to the increasing difficulty of the prediction task. However, as shown in the Additional files 3: Table S5 and S6, using the soft voting ensemble strategy in the second stage also results in an 11–14% improvement which further validates the effectiveness and superiority of the soft voting strategy.

### t-SNE visualization of extracted features

**Fig. 4:**
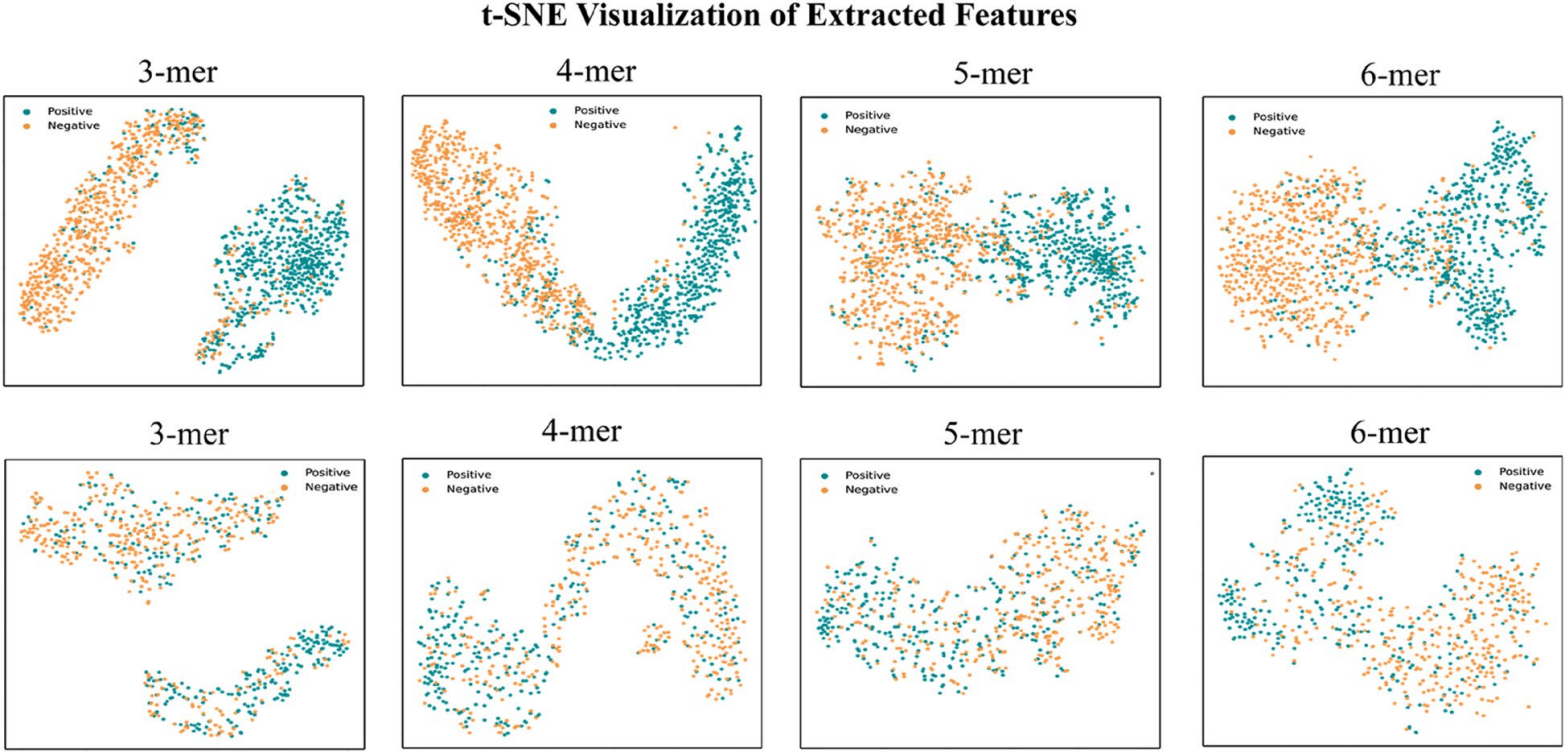
t-SNE visualization of extracted features by different base predictors. The top four representing the promoter identification stage and the bottom four representing the promoter strength prediction stage

To intuitively compare and analyze how different base predictors extract features from biological sequences, we used t-SNE for visualizing the extracted sequence features. Specifically, we extracted the 768-dimensional features from the twelfth encoding layer of the model and reduced them to two dimensions using t-SNE for easy analysis of how the model classifies sequence data. The experimental results are shown in Fig. 4.

From the visualization, we can clearly see that during the promoter identification stage, all base predictors are able to separate positive samples from negative samples. During the promoter strength prediction stage, the model still overall distinguishes between positive and negative samples, but the proportion of misclassified samples increases. This is due to the increased difficulty of the prediction task. It is worth noting that regardless of the promoter identification or strength prediction stage, the 3-mer base predictor is able to separate all samples into two distinct classes, with a larger distance between samples of different classes. On the other hand, the remaining base predictors only roughly separate samples into two classes, with samples of different classes being closer together. This phenomenon further explains the distribution observed in the kernel density estimation plot in Fig. 3B. Because the 3-mer base predictor can clearly separate all samples into two classes, its predicted probability values are more spread out, and the overlapping region between positive and negative samples is relatively small. In contrast, the other base predictors exhibit samples of different classes being close to each other, which indicates sensitivity to the learned boundary function. Consequently, this leads to a minimal difference in predicted probability values between positive and negative samples, resulting in a larger overlapping region observed between them in the kernel density estimation plot. Therefore, the t-SNE visualization provide further insights into how different base predictors extract and classify sequence features in the stage of promoter identification and strength prediction.

## Conclusion

The primary work of this research is to introduce a two-stage prediction framework aimed at the identification of promoters and the subsequent prediction of their strengths. To achieve this, we adopt a multi-scale feature extraction approach by segmenting the DNA sequence into different tokens, including 3-mer, 4-mer, 5-mer, and 6-mer, which are then inputted into the pre-trained model DNABERT. The results from different base predictors are integrated using a soft voting method. Through attention mechanisms analysis, it was discovered that the model effectively integrates both local and global information of the promoter sequence. Compared to other traditional deep learning-based methods, our model demonstrates better benchmark performance and generalization ability. Additionally, a range of analyses conducted during this study demonstrate that our predictions surpass those of existing state-of-the-art predictors, particularly in regards to promoter identification and strength prediction. This method contributes to our understanding of the prediction mechanisms of pre-trained models in the context of biological sequences and effectively addresses bioinformatics problems with outstanding performance.

## Supplementary Information

**Additional file 1: Table S1.** Detailed hyperparameter settings of four base predictors on promoter identification. Table S2. Detailed hyperparameter settings of four base predictors on promoter strength prediction.

**Additional file 2: Table S3.** Performance comparison of integrating different base predictors on promoter identification. Table S4. Performance comparison of integrating different base predictors on promoter strength prediction.

**Additional file 3: Table S5.** Comparison with the baseline predictors on promoter classification. Table S6. Comparison with the baseline predictors on promoter strength prediction.

## Data Availability

Data and source code for msBERT-Promoter can be found in a GitHub repository: https://github.com/liyazi712/msBERT-Promoter [54].

## Abbreviations

SVM: Support vector machine
PseKNC: Pseudo k-tuple nucleotide composition
PCSF: Position-correlation scoring function
mRMR: Minimum redundancy maximum relevance
BERT: Bidirectional Encoder Representations from Transformers
NLP: Natural language processing
RBPs: RNA-binding proteins
SHAP: Shapley Additive exPlanations
ACC: Accuracy
MCC: Matthews correlation coefficient
AUC: Area under curve
Sn: Sensitivity
Sp: Specificity
NSP: Next sentence prediction
MLM: Masked language model
ROC: Receiver operating characteristic
CNN: Convolutional neural network
RNN: Recurrent neural network
