# Supplementary material for: msBERT-Promoter: a multi-scale ensemble predictor based on BERT pre-trained model for the two-stage prediction of DNA promoters and their strengths
Source: BMC Biol. 2024 May 30;22:126. doi: 10.1186/s12915-024-01923-z (PMC11555825; doi:10.1186/s12915-024-01923-z)
Supplement: Supplementary file 1 — Additional file 1: Table S1. Detailed hyperparameter settings of four base predictors on promoter identification. Table S2. Detailed hyperparameter settings of four base predictors on promoter strength prediction. [file 12915_2024_1923_MOESM1_ESM.docx]

**Table S1**. Detailed hyperparameter settings of four base predictors on promoter identification.

| k-mer | learning rate | batch size | seq length | dropout | warmup | weight decay |
| --- | --- | --- | --- | --- | --- | --- |
| 3-mer | 2.00E-06 | 32 | 81 | 0.1 | 0.1 | 0.01 |
| 4-mer | 2.00E-05 | 64 | 81 | 0.1 | 0.1 | 0.01 |
| 5-mer | 5.00E-05 | 32 | 81 | 0.1 | 0.1 | 0.01 |
| 6-mer | 4.00E-05 | 32 | 81 | 0.1 | 0.1 | 0.01 |

**Table S2**. Detailed hyperparameter settings of four base predictors on promoter strength prediction.

| k-mer | learning rate | batch size | seq length | dropout | warmup | weight decay |
| --- | --- | --- | --- | --- | --- | --- |
| 3-mer | 2.00E-06 | 64 | 81 | 0.1 | 0.1 | 0.01 |
| 4-mer | 2.00E-05 | 32 | 81 | 0.1 | 0.1 | 0.01 |
| 5-mer | 5.00E-05 | 64 | 81 | 0.1 | 0.1 | 0.01 |
| 6-mer | 4.00E-05 | 32 | 81 | 0.1 | 0.1 | 0.01 |
