# Supplementary material for: msBERT-Promoter: a multi-scale ensemble predictor based on BERT pre-trained model for the two-stage prediction of DNA promoters and their strengths
Source: BMC Biol. 2024 May 30;22:126. doi: 10.1186/s12915-024-01923-z (PMC11555825; doi:10.1186/s12915-024-01923-z)
Supplement: Supplementary file 2 — Additional file 2: Table S3. Performance comparison of integrating different base predictors on promoter identification. Table S4. Performance comparison of integrating different base predictors on promoter strength prediction. [file 12915_2024_1923_MOESM2_ESM.docx]

**Table S3**. Performance comparison of integrating different base predictors on promoter identification.

|  | Sn | Sp | Acc | AUC | MCC |
| --- | --- | --- | --- | --- | --- |
| 3mer+4mer+5mer+6mer | **0.9728** | **0.9509** | **0.9616** | **0.9943** | **0.9234** |
| 3mer+4mer+5mer | 0.9583 | 0.8906 | 0.9217 | 0.9837 | 0.8461 |
| 3mer+4mer+6mer | 0.9523 | 0.9176 | 0.9343 | 0.9855 | 0.8692 |
| 3mer+5mer+6mer | 0.9620 | 0.9032 | 0.9306 | 0.9864 | 0.8631 |
| 4mer+5mer+6mer | 0.9694 | 0.8977 | 0.9306 | 0.9875 | 0.8641 |
| 3mer+4mer | 0.8914 | 0.8755 | 0.8833 | 0.9633 | 0.7668 |
| 3mer+5mer | 0.8976 | 0.8714 | 0.8840 | 0.9612 | 0.7685 |
| 3mer+6mer | 0.9041 | 0.8809 | 0.8922 | 0.9684 | 0.7847 |
| 4mer+5mer | 0.9313 | 0.8709 | 0.8988 | 0.9629 | 0.7999 |
| 4mer+6mer | 0.9315 | 0.9067 | 0.9188 | 0.9695 | 0.8379 |
| 5mer+6mer | 0.9489 | 0.8860 | 0.9151 | 0.9697 | 0.8325 |
| 3mer | 0.8718 | 0.8567 | 0.8641 | 0.9200 | 0.7284 |
| 4mer | 0.8641 | 0.8057 | 0.8323 | 0.9070 | 0.6672 |
| 5mer | 0.8854 | 0.7776 | 0.8227 | 0.8943 | 0.6542 |
| 6mer | 0.8896 | 0.8340 | 0.8597 | 0.9230 | 0.7215 |

**Table S4**. Performance comparison of integrating different base predictors on promoter strength prediction.

|  | Sn | Sp | Acc | AUC | MCC |
| --- | --- | --- | --- | --- | --- |
| 3mer+4mer+5mer+6mer | **0.8138** | 0.7861 | **0.7979** | **0.8742** | **0.5946** |
| 3mer+4mer+5mer | 0.8024 | 0.7271 | 0.7552 | 0.8360 | 0.5130 |
| 3mer+4mer+6mer | 0.7586 | 0.7448 | 0.7507 | 0.8445 | 0.4990 |
| 3mer+5mer+6mer | 0.7414 | **0.8152** | 0.7773 | 0.8532 | 0.5573 |
| 4mer+5mer+6mer | 0.8118 | 0.7568 | 0.7788 | 0.8421 | 0.5580 |
| 3mer+4mer | 0.7470 | 0.6900 | 0.7109 | 0.7895 | 0.4220 |
| 3mer+5mer | 0.7083 | 0.7322 | 0.7212 | 0.7993 | 0.4399 |
| 3mer+6mer | 0.6710 | 0.7898 | 0.7227 | 0.8019 | 0.4577 |
| 4mer+5mer | 0.7397 | 0.6789 | 0.7006 | 0.7851 | 0.4018 |
| 4mer+6mer | 0.7500 | 0.7122 | 0.7271 | 0.8081 | 0.4527 |
| 5mer+6mer | 0.6886 | 0.7622 | 0.7242 | 0.8109 | 0.4513 |
| 3mer | 0.6319 | 0.6790 | 0.6563 | 0.7139 | 0.3112 |
| 4mer | 0.7195 | 0.6713 | 0.6888 | 0.7333 | 0.3765 |
| 5mer | 0.6679 | 0.6683 | 0.6681 | 0.7210 | 0.3316 |
| 6mer | 0.6328 | 0.7415 | 0.6799 | 0.7530 | 0.3716 |
