# Supplementary material for: msBERT-Promoter: a multi-scale ensemble predictor based on BERT pre-trained model for the two-stage prediction of DNA promoters and their strengths
Source: BMC Biol. 2024 May 30;22:126. doi: 10.1186/s12915-024-01923-z (PMC11555825; doi:10.1186/s12915-024-01923-z)
Supplement: Supplementary file 3 — Additional file 3: Table S5. Comparison with the baseline predictors on promoter classification. Table S6. Comparison with the baseline predictors on promoter strength prediction. [file 12915_2024_1923_MOESM3_ESM.docx]

**Table S5**. Comparison with the baseline predictors on promoter classification

|  | Sn | Sp | Acc | AUC | MCC |
| --- | --- | --- | --- | --- | --- |
| Transformer | 0.755 | 0.787 | 0.770 | 0.843 | 0.544 |
| BERT_DPCNN | **0.820** | 0.844 | 0.832 | 0.888 | **0.664** |
| GCN | 0.718 | 0.831 | 0.776 | 0.849 | 0.554 |
| TEXT_GCN | 0.448 | 0.580 | 0.516 | 0.498 | 0.029 |
| GAT | 0.695 | 0.851 | 0.776 | 0.848 | 0.555 |
| DNN | 0.762 | 0.820 | 0.790 | 0.852 | 0.583 |
| LSTM | 0.796 | 0.830 | 0.813 | 0.874 | 0.626 |
| GRU | 0.753 | 0.878 | 0.814 | 0.876 | 0.635 |
| DNABERT | 0.810 | **0.917** | **0.866** | **0.895** | 0.661 |

**Table S6**. Comparison with the baseline predictors on promoter strength prediction.

|  | Sn | Sp | Acc | AUC | MCC |
| --- | --- | --- | --- | --- | --- |
| Transformer | 0.552 | 0.577 | 0.565 | 0.585 | 0.130 |
| BERT_DPCNN | 0.472 | 0.664 | 0.573 | 0.589 | 0.139 |
| GCN | 0.640 | 0.372 | 0.506 | 0.495 | 0.013 |
| TEXT_GCN | 0.621 | 0.489 | 0.555 | 0.520 | 0.111 |
| GAT | 0.265 | 0.751 | 0.508 | 0.491 | 0.019 |
| DNN | 0.495 | 0.646 | 0.576 | 0.602 | 0.143 |
| LSTM | 0.422 | 0.674 | 0.556 | 0.541 | 0.099 |
| GRU | 0.466 | 0.632 | 0.555 | 0.546 | 0.100 |
| DNABERT | **0.709** | **0.753** | **0.732** | **0.789** | **0.463** |
